# Response to a Large Polio Outbreak in a Setting of Conflict — Middle East, 2013–2015

**DOI:** 10.15585/mmwr.mm6608a6

**Published:** 2017-03-03

**Authors:** Chukwuma Mbaeyi, Michael J. Ryan, Philip Smith, Abdirahman Mahamud, Noha Farag, Salah Haithami, Magdi Sharaf, Jaume C. Jorba, Derek Ehrhardt

**Affiliations:** ^1^Global Immunization Division, Center for Global Health, CDC; ^2^Polio Eradication Department, World Health Organization, Geneva, Switzerland; ^3^Division of Viral Diseases, National Center for Immunization and Respiratory Diseases, CDC.

As the world advances toward the eradication of polio, outbreaks of wild poliovirus (WPV) in polio-free regions pose a substantial risk to the timeline for global eradication. Countries and regions experiencing active conflict, chronic insecurity, and large-scale displacement of persons are particularly vulnerable to outbreaks because of the disruption of health care and immunization services (*1*). A polio outbreak occurred in the Middle East, beginning in Syria in 2013 with subsequent spread to Iraq (*2*). The outbreak occurred 2 years after the onset of the Syrian civil war, resulted in 38 cases, and was the first time WPV was detected in Syria in approximately a decade (*3*,*4*). The national governments of eight countries designated the outbreak a public health emergency and collaborated with partners in the Global Polio Eradication Initiative (GPEI) to develop a multiphase outbreak response plan focused on improving the quality of acute flaccid paralysis (AFP) surveillance* and administering polio vaccines to >27 million children during multiple rounds of supplementary immunization activities (SIAs).^†^ Successful implementation of the response plan led to containment and interruption of the outbreak within 6 months of its identification. The concerted approach adopted in response to this outbreak could serve as a model for responding to polio outbreaks in settings of conflict and political instability.

## Outbreak Detection and Epidemiology

Detection of the Middle East outbreak depended upon systems for AFP surveillance in the affected countries, including the World Health Organization's (WHO’s) Early Warning, Alert and Response Network (EWARN)^§^, through which the outbreak was identified in October 2013. The nonpolio AFP (NPAFP) and stool adequacy rates served as indicators for assessing the ability of the affected countries to detect polio cases and also to determine when the outbreak had been interrupted.

Among countries that reported polio cases, the NPAFP rate in Syria in 2012 was 1.4 cases per 100,000 persons aged <15 years, below the recommended benchmark of ≥2. The NPAFP rate for Syria improved, increasing to 1.7 cases per 100,000 persons in 2013, the year the outbreak was detected, and to 4.0 and 3.0 in 2014 and 2015, respectively (Table). In Iraq, the NPAFP rate ranged from 3.1 to 4.0 during 2012–2015; estimates of NPAFP rates in Syria and Iraq might, however, be inaccurate because of the large-scale conflict-related displacement of persons and the attendant impact on target population estimates. Among countries at risk, NPAFP rates were suboptimal in Jordan at the onset, but improved over the course of the outbreak, increasing from 1.4 in 2013 to 3.2 in 2015. Despite incremental improvements, NPAFP rates remained <2 in Turkey over the course of the outbreak, and rates declined in Palestine from 2.2 in 2013 to 1.2 in 2014 before improving to 2.2 in 2015. All other countries involved in the response achieved recommended benchmarks.

**TABLE:** Acute flaccid paralysis (AFP) surveillance indicators and outbreak response activities by country and year — eight countries in the Middle East, 2012–2015

Year/Activity	Country							
	Egypt	Iran	Iraq	Jordan	Lebanon	Palestine	Syria	Turkey
**2012**								
Nonpolio AFP rate*	3.9	3.5	3.8	1.5	2.2	1.3	1.4	0.9
AFP cases with adequate specimens (%)	92	92	90	84	50	95	84	80
**2013**								
Nonpolio AFP rate*	3	4	3.1	1.4	2.2	2.2	1.7	1.2
AFP cases with adequate specimens (%)	92	96	84	91	45	95	68	76
SIAs	2 NIDs	—^†^	2 NIDs	2 NIDs	2 NIDs	1 NID	2 NIDs	2 SNIDs
			1 SNID					
**2014**								
Nonpolio AFP rate*	2.9	4.2	4	2.5	2.7	1.2	4	1.5
AFP cases with adequate specimens (%)	93	96	89	97	70	90	84	77
SIAs	2 NIDs	2 SNIDs	7 NIDs	3 NIDs	4 NIDs	1 NID	8 NIDs	5 SNIDs
	1 SNID		3 SNIDs	2 SNIDs	3 SNIDs		1 SNID	
**2015**								
Nonpolio AFP rate*	3	4.3	3.6	3.2	5.2	2.2	3	1.7
AFP cases with adequate specimens (%)	94	97	82	97	84	92	90	82
SIAs	1 NID	2 SNIDs	5 NIDs	1 SNID	2 SNIDs	—^†^	4 NIDs	2 SNIDs
	2 SNIDs						2 SNIDs	

**Abbreviations**: NIDs = national immunization days; SIAs = supplemental immunization activities; SNIDs = subnational immunization days.

* Cases per 100,000 children aged <15 years (target: ≥2 per 100,000).

^†^ No NIDs or SNIDs conducted for the year.

Rates of stool specimen adequacy (i.e., receipt of two stool specimens collected at least 24 hours apart within 14 days of paralysis onset and properly shipped to the laboratory) in Syria increased from 68% in 2013 to 90% in 2015; in Iraq, rates of stool specimen adequacy exceeded the benchmark of ≥80% in each year during 2012–2015. Lebanon showed substantial gaps in stool specimen adequacy before and during the outbreak with rates ranging from 45% to 70% during 2012–2014, but the rate improved to 84% in 2015.

**FIGURE 1:**
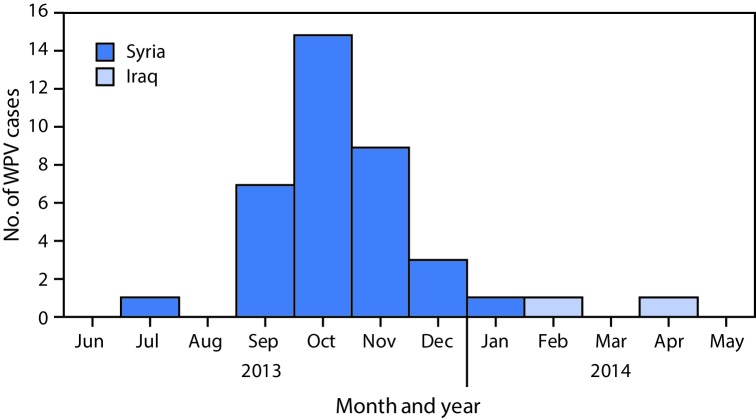
Number of cases of wild poliovirus type 1 (WPV1), by month and year of paralysis onset — Syria and Iraq, 2013–2014

A total of 38 WPV type 1 cases were reported during the outbreak, with dates of paralysis onset ranging from July 14, 2013 for the index case (Aleppo, Syria) to April 7, 2014 for the last confirmed case (Baghdad, Iraq). The outbreak was virologically confirmed in October 2013. Of the 38 cases reported, 36 occurred in Syria and two occurred in Iraq (Figure 1). Approximately two thirds (24 of 38) of reported cases occurred in male children and 74% of cases occurred in children aged <2 years. Fifty-eight percent of children with polio had never received oral poliovirus vaccine (OPV) either through routine or supplementary immunization (i.e., zero-dose children), and an additional 37% of children with polio had received ≤3 OPV doses. The remaining 5% of children with polio had received 3 OPV doses.

**FIGURE 2:**
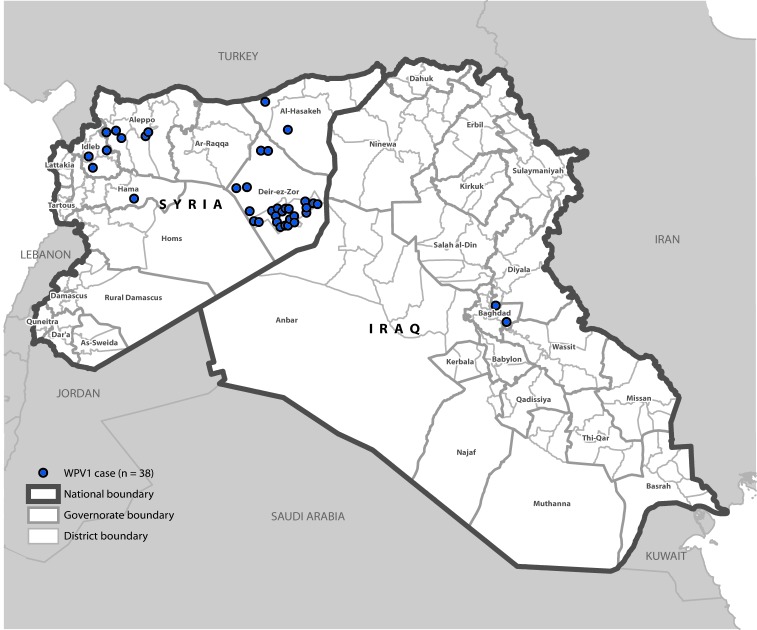
Cases of wild poliovirus type 1 (WPV1) — Syria and Iraq, 2013–2014* * Each dot represents one case. Dots are randomly placed within second administrative units.

Thirty-five of the 36 polio cases in Syria were reported during 2013 and the last identified case had paralysis onset in January 2014. A breakdown of cases by governorate (Figure 2) indicates that 25 (69%) cases were reported from Deirez-Zour, five from Aleppo, three from Edleb, two from Hasakeh, and one from Hama. The two cases reported from Iraq occurred in February and April 2014; both were from Baghdad-Resafa Governorate. Both cases were related by genetic sequencing and were closely linked to WPV circulating in Syria. Genetic sequencing indicated virus circulation might have begun a year earlier somewhere in the Middle East, coincident with identification of WPV-positive environmental samples in Egypt in December 2012 (*5*). The implicated viral strain was genetically linked to strains circulating in Pakistan (*6*).

## Outbreak Response Plan Development

Eight countries in the region (Egypt, Iran, Iraq, Jordan, Lebanon, Palestine, Syria, and Turkey) developed a concerted Middle East polio outbreak response plan, which was updated during the course of the outbreak. Countries were grouped into two areas: 1) countries with poliovirus transmission (Syria and Iraq), and 2) countries at significant risk for poliovirus importation based on geographic proximity and influx of displaced persons from the outbreak zone (Egypt, Iran, Jordan, Lebanon, Palestine, and Turkey). The strategic response in these areas occurred in three phases. Phase I (October 2013–April 2014) focused on interrupting WPV transmission and halting spread of the virus beyond the affected countries. Phase II (May 2014–January 2015) identified areas at high risk for poliovirus importation and circulation based on stipulated criteria, including presence of refugees and mobile populations, security-compromised areas, districts with low vaccination coverage, and geographically hard-to-reach communities. These areas were prioritized for SIAs and intensified surveillance activities. Phase III (February–October 2015) was aimed at further boosting population immunity against polio through strengthened routine immunization systems and SIAs.

**Immunization Coverage**. Conflict in Syria and Iraq in the years preceding and following the outbreak led to steep declines in routine vaccination coverage among children in both countries, in contrast to most other countries in the Middle East where coverage remained high. Estimated national routine vaccination coverage of infants in Syria with 3 doses of oral poliovirus vaccine (OPV3) declined from preconflict levels of 83% in 2010 to 47%–52% during 2012–2014.^¶^ Estimates of coverage in Iraq were ≤70% and coverage in Lebanon was 75% during 2012–2014. All other countries involved in the response had coverage levels of >90% during 2012–2014.

In response to the Middle East polio outbreak, >70 SIAs were conducted during October 2013–December 2015. SIAs targeted approximately 27 million children aged <5 years in eight countries and were conducted using trivalent (types 1, 2, and 3) and bivalent (types 1 and 3) OPV. Strategies used during the campaigns included fixed-post (health facility), house-to-house visits, transit-point vaccination, and deployment of mobile teams to vulnerable populations and geographically hard-to-reach areas. Strategies were tailored to the unique sociocultural context of each country involved in the response.

**Implementation of outbreak response plan.** Following identification of the outbreak, Syria conducted two rounds of national immunization days (NIDs) in November and December 2013, eight NIDs and one round of subnational immunization days (SNIDs) in 2014, and four NIDs and two SNIDs in 2015 (Table). Postcampaign monitoring coverage estimates improved from 79% in December 2013 to 93% in March 2014, with coverage levels ≥88% during a majority of the campaigns. Iraq held 14 NIDs and four SNIDs as part of the response, with postcampaign monitoring coverage levels ranging from 86% to 94% during 2014. Egypt, Iran, Jordan, Lebanon, Palestine, and Turkey conducted two to 11 vaccination campaigns.

Active conflict in many parts of Syria and some parts of Iraq limited access for vaccination activities during the course of the response. Negotiations with local authorities and engagement of community leaders enabled implementation of a limited number of vaccination campaigns in some conflict-affected areas, but it was difficult to monitor these campaigns, or generate reliable data on the quality of response activities. Egypt, Iraq, Jordan, Lebanon, and Turkey received large numbers of Syrian refugees (*7*), which placed significant strain on their health care resources and increased costs of implementing outbreak response activities. Refugees aged <15 years living in camps in Jordan were vaccinated against polio upon registration and entry, and during special vaccination campaigns held in camps.

In assessing the effect of outbreak response activities, the vaccination status of nonpolio AFP cases in children aged 6–59 months in Syria and Iraq was reviewed. The proportion of NPAFP cases among children aged 6–59 months who were reported to have received ≥3 doses of OPV in Syria rose from 82% in 2013 to 94% in 2015, but remained at 93% among Iraqi children of the same age group during 2013–2015. The proportion of children aged 6–59 months with NPAFP who had never received OPV, or any other form of polio vaccination, decreased from 9% in 2013 to 2% in 2015 in Syria, but increased slightly from 1% to 3% in Iraq during the same period.

## Discussion

The Middle East polio outbreak occurred within an extremely challenging setting, given the ongoing civil war in Syria and conflict in several parts of Iraq. The near collapse of the health care system in conflict-affected parts of Syria resulted in plummeting levels of routine vaccination coverage that left many children born after the start of the civil war unimmunized or underimmunized against polio, and set the stage for the spread of poliovirus following importation within this age group and beyond.

Actions were taken to mitigate the risk for a polio outbreak in Syria when WPV-positive environmental isolates were identified in Egypt late in 2012. AFP surveillance activities in Syria, including in opposition-controlled areas, were intensified through WHO's EWARN system, and polio vaccination campaigns were conducted in all of Syria’s governorates by January 2013 (*6*). However, the cohort of children born during the conflict remained vulnerable to a polio outbreak because of steep declines in routine polio vaccination coverage.

After a cluster of WPV cases was detected in Deirez-Zour Governorate, the government of Syria immediately declared the outbreak a public health emergency. A multicountry response plan was developed to contain and interrupt the outbreak, which was effectively contained within 6 months from the time of its identification. Improvements in AFP surveillance performance indicators in the outbreak-affected countries provided a basis for WHO to declare the outbreak over in 2015. In addition to intensified surveillance and immunization activities, the response owed its success in large part to the level of collaboration and concerted approach adopted by eight national governments in the region. Another factor contributing to the success of the response was that high routine immunization coverage in many countries in the region, coupled with high prewar vaccination coverage in Syria, limited the population of vulnerable persons to mostly children born after the onset of the civil war.

With the attention of GPEI focused on the final push to interrupt indigenous WPV transmission in the remaining three polio-endemic countries (*8*–*10*), vigilance must be maintained in the Middle East and other conflict-affected areas to forestall the risk for new WPV outbreaks. In the event of a new outbreak, the Middle East polio outbreak response provides a model for an effective response within challenging settings.

**Summary**

**What is already known about this topic?**

Afghanistan, Nigeria, and Pakistan are the only three countries that have never interrupted endemic transmission of wild poliovirus (WPV). Continued WPV circulation in these countries poses a risk for polio outbreaks in polio-free regions of the world, especially in countries experiencing conflict and insecurity, with attendant disruption of health care and immunization services.

**What is added by this report?**

A WPV outbreak occurred in Syria and Iraq during 2013–2014 after importation of a poliovirus strain circulating in Pakistan. The outbreak represented the first occurrence of polio cases in both countries in approximately a decade, and resulted in 38 polio cases, including 36 in Syria and two in Iraq. Development and implementation of an integrated response plan for strengthening acute flaccid paralysis surveillance and synchronized mass vaccination campaigns by eight national governments in the Middle East facilitated interruption of the outbreak within 6 months of its identification.

**What are the implications for public health practice?**

Countries experiencing active conflict and chronic insecurity are at increased risk for polio outbreaks because of political instability and population displacement hindering delivery of immunization services. Adoption of a concerted approach to planning and implementing response activities, with involvement of more stable neighboring countries, could serve as a useful model for polio outbreak response in areas affected by conflict, as exemplified by the Middle East polio outbreak response.

## Footnotes

* The quality of acute flaccid paralysis (AFP) surveillance is monitored by performance indicators that include 1) the detection rate of nonpolio AFP (NPAFP) cases, and 2) the proportion of AFP cases with adequate stool specimens. World Health Organization (WHO) operational targets for countries with endemic poliovirus transmission are an NPAFP detection rate of ≥2 cases per 100,000 population aged <15 years, and adequate stool specimen collection from ≥80% of AFP cases, in which two specimens are collected ≥24 hours apart, both within 14 days of paralysis onset, and shipped on ice or frozen packs to a WHO-accredited laboratory, arriving in good condition (without leakage or desiccation).

† Mass campaigns conducted for a brief period (days to weeks) in which 1 dose of oral poliovirus vaccine is administered to all children aged <5 years, regardless of vaccination history. Campaigns are conducted nationally or subnationally (i.e., in portions of the country).

§
http://apps.who.int/iris/bitstream/10665/70812/1/WHO_HSE_GAR_DCE_2012_1_eng.pdf .

¶
http://apps.who.int/immunization_monitoring/globalsummary .
